# Supplementary material for: P-TRAP: a Panicle Trait Phenotyping tool
Source: BMC Plant Biol. 2013 Aug 29;13:122. doi: 10.1186/1471-2229-13-122 (PMC3848748; doi:10.1186/1471-2229-13-122)
Supplement: Additional file 7 — P-TRAP source code. The Netbeans project that contains the source code of the application. [file 1471-2229-13-122-S7.zip › javadoc.zip!/javadoc/org-bioalg-imageprocessor/org/bioalg/imageprocessor/RGBPixel.html]

RGBPixel (Image Processor)


JavaScript is disabled on your browser.


- Package
- Class
- Tree
- Deprecated
- Index
- Help

*org.bioalg.imageprocessor 1.0*

- Prev Class
- Next Class

- Frames
- No Frames

- All Classes

- Summary:
- Nested |
- Field |
- Constr |
- Method

- Detail:
- Field |
- Constr |
- Method


org.bioalg.imageprocessor

## Class RGBPixel

- java.lang.Object
- - org.bioalg.imageprocessor.RGBPixel

- ---

    

  ```
  public class RGBPixel
  extends java.lang.Object
  ```

  The Red Green Blue model of the pixel.

  Author:
  :   faroqal-tam

- - ### Constructor Summary

    Constructors

    Constructor and Description

    `RGBPixel()`

    `RGBPixel(int rgb)`

    `RGBPixel(int r,
    int g,
    int b)`
  - ### Method Summary

    Methods

    | Modifier and Type | Method and Description |
    | --- | --- |
    | `double` | `distance(RGBPixel rgbp)` |
    | `boolean` | `equals(java.lang.Object obj)` |
    | `int` | `getB()` |
    | `int` | `getG()` |
    | `int` | `getR()` |
    | `void` | `setB(int b)` |
    | `void` | `setG(int g)` |
    | `void` | `setR(int r)` |
    | `int` | `toInteger()` |
    | `java.lang.String` | `toString()` |

    - ### Methods inherited from class java.lang.Object

      `clone, finalize, getClass, hashCode, notify, notifyAll, wait, wait, wait`

- - ### Constructor Detail


    - #### RGBPixel

      ```
      public RGBPixel()
      ```


    - #### RGBPixel

      ```
      public RGBPixel(int r,
              int g,
              int b)
      ```


    - #### RGBPixel

      ```
      public RGBPixel(int rgb)
      ```
  - ### Method Detail


    - #### toInteger

      ```
      public int toInteger()
      ```


    - #### getR

      ```
      public int getR()
      ```


    - #### setR

      ```
      public void setR(int r)
      ```


    - #### getG

      ```
      public int getG()
      ```


    - #### setG

      ```
      public void setG(int g)
      ```


    - #### getB

      ```
      public int getB()
      ```


    - #### setB

      ```
      public void setB(int b)
      ```


    - #### equals

      ```
      public boolean equals(java.lang.Object obj)
      ```

      **Overrides:**
      :   `equals` in class `java.lang.Object`


    - #### distance

      ```
      public double distance(RGBPixel rgbp)
      ```


    - #### toString

      ```
      public java.lang.String toString()
      ```

      **Overrides:**
      :   `toString` in class `java.lang.Object`


- Package
- Class
- Tree
- Deprecated
- Index
- Help

*org.bioalg.imageprocessor 1.0*

- Prev Class
- Next Class

- Frames
- No Frames

- All Classes

- Summary:
- Nested |
- Field |
- Constr |
- Method

- Detail:
- Field |
- Constr |
- Method
